# Spontaneous decays of magneto-elastic excitations in non-collinear antiferromagnet (Y,Lu)MnO_3_

**DOI:** 10.1038/ncomms13146

**Published:** 2016-10-19

**Authors:** Joosung Oh, Manh Duc Le, Ho-Hyun Nahm, Hasung Sim, Jaehong Jeong, T. G. Perring, Hyungje Woo, Kenji Nakajima, Seiko Ohira-Kawamura, Zahra Yamani, Y. Yoshida, H. Eisaki, S. -W. Cheong, A. L. Chernyshev, Je-Geun Park

**Affiliations:** 1 Center for Correlated Electron Systems, Institute for Basic Science, Seoul 08826, Korea; 2 Department of Physics and Astronomy, Seoul National University, Kwanak-Gu, Seoul 08826, Korea; 3 ISIS Facility, STFC Rutherford Appleton Laboratory, Didcot OX11 0QX, UK; 4 Department of Physics, Brookhaven National Laboratory, Upton, New York 11973, USA; 5 Materials and Life Science Division, J-PARC Center, Japan Atomic Energy Agency, Tokai, Ibaraki 319-1195, Japan; 6 Chalk River Laboratories, National Research Council, Chalk River, Ontario, Canada K0J 1J0; 7 National Institute of Advanced Industrial Science and Technology, Tsukuba, Ibaraki 305-8565, Japan; 8 Department of Physics and Astronomy, and Rutgers Center for Emergent Materials, Rutgers University, Piscataway, New Jersey 08854, USA; 9 Department of Physics and Astronomy, University of California, Irvine, California 92697, USA

## Abstract

Magnons and phonons are fundamental quasiparticles in a solid and can be coupled together to form a hybrid quasi-particle. However, detailed experimental studies on the underlying Hamiltonian of this particle are rare for actual materials. Moreover, the anharmonicity of such magnetoelastic excitations remains largely unexplored, although it is essential for a proper understanding of their diverse thermodynamic behaviour and intrinsic zero-temperature decay. Here we show that in non-collinear antiferromagnets, a strong magnon–phonon coupling can significantly enhance the anharmonicity, resulting in the creation of magnetoelastic excitations and their spontaneous decay. By measuring the spin waves over the full Brillouin zone and carrying out anharmonic spin wave calculations using a Hamiltonian with an explicit magnon–phonon coupling, we have identified a hybrid magnetoelastic mode in (Y,Lu)MnO_3_ and quantified its decay rate and the exchange-striction coupling term required to produce it.

## Abstract (web summary)

The properties of magnetic, crystalline solids can be described in terms of quantum particles of spin-wave and lattice-vibration energy, known as magnons and phonons respectively. Here, the authors show that strong magnon-phonon coupling in a noncollinear antiferromagnet can create magnetoelastic excitations.

## Graphical abstract

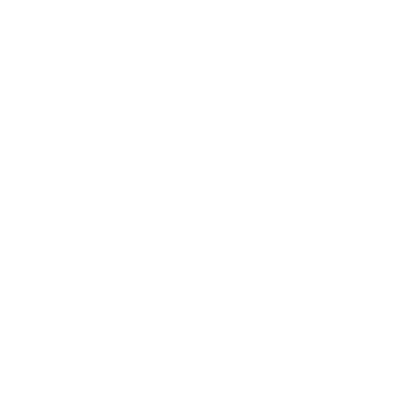

Spin and lattice constitute two of the four fundamental degrees of freedom in the solid: the other two are charge and orbital. In linearized models that account for many current understandings of the solid, excitations of spin and lattice, magnon of spin waves and phonon of lattice vibrations, are two principal examples of such quasiparticles^1^. Although there have been some experimental observations of cross-coupling between magnon and phonon, it is rather rare to actually observe and, more importantly, quantify the magnon–phonon coupling for real materials^2^,^3^. Nevertheless, it is essential for a proper understanding of their diverse thermodynamic behaviour and intrinsic zero-temperature decay^4^. Furthermore, it is generally believed that the magnon–phonon coupling is important for materials such as multiferroic compounds, geometrically frustrated systems, spin-Peierls systems and Invar materials, to name only a few^5^,^6^,^7^,^8^.

Quasiparticles like magnon and phonon, the cornerstone of modern condensed matter physics, are fundamentally the byproducts of linearized theories that ignore all the higher-order terms than quadratic terms and neglect any conceivable interaction among the quasiparticles themselves. As such, they are considered to be stable, except for very few exceptions. For example, for classical spin systems without strong quantum fluctuations, magnon breakdown is thought to be unlikely for most of purposes. Therefore, observing and understanding how the otherwise stable quasiparticles break down in these unusual cases are the central theme of condensed matter physics.

One route leading to the breakdown of magnon and phonon is the cubic anharmonicities. Despite the general belief that this nonlinear magnon(-magnon) interaction is rather weak in real materials, recent insights gained mainly through theoretical studies suggest that things should be drastically different for certain cases, namely for non-collinear antiferromagnetic structures^4^. Unlike collinear magnetic structures that forbid the cubic anharmonicities, it was shown that such interaction is in principle allowed for non-collinear magnetic structures, such as the canonical 120° spin pattern in a two-dimensional (2D) triangular lattice. There have since been several experimental reports^9^,^10^ supporting these theoretical postulates. Nevertheless, the full details of the nonlinear interaction still need to be worked out, especially from experiments. We should also point out that this non-collinear magnetic order, in principle, allows a hitherto forbidden magnon–phonon coupling that has been less recognized among the community: the first-order variation of the exchange energy with respect to transverse spin fluctuations is non-zero for a non-collinear magnetic order^11^. As the O(3) symmetry is completely broken in the non-collinear ordered ground state of spins, coupling to phonons necessarily generates a coupling in which a magnon can convert directly into a phonon and vice versa. This is in contrast to the spin–lattice coupling in more conventional, collinear magnets, where the coupling usually respects the parity, which necessarily conserves the number of magnons or allows creation (annihilation) of them only in pairs^12^.

Here we report the direct observation of the magnon–phonon coupling and the spontaneous decay of magneto-elastic excitations in the triangular antiferromagnets (Y,Lu)MnO_3_. The full magnetic excitation spectra measured by inelastic neutron-scattering experiments show clear deviations from the linear spin wave theory without the magnon–phonon coupling: an additional mode at high energies and the downward shift of the bottom mode at the Brillouin zone boundary. This is the most direct and stark evidence of the linear coupling of magnons and phonons, which, in turn, leads to enhancement of the anharmonic effects. We demonstrate that these experimental anomalies can only be fully resolved by incorporating the magnon–phonon coupling and carrying out the nonlinear spin wave analysis. We further reveal that the magneto-elastic excitation leads to significant broadening of the magnon spectra at the zone boundaries, originating from the decay of the magneto-elastic excitations into the two-magnon continuum.

## Results

### Failure of standard spin waves calculation

**Figure 1:**
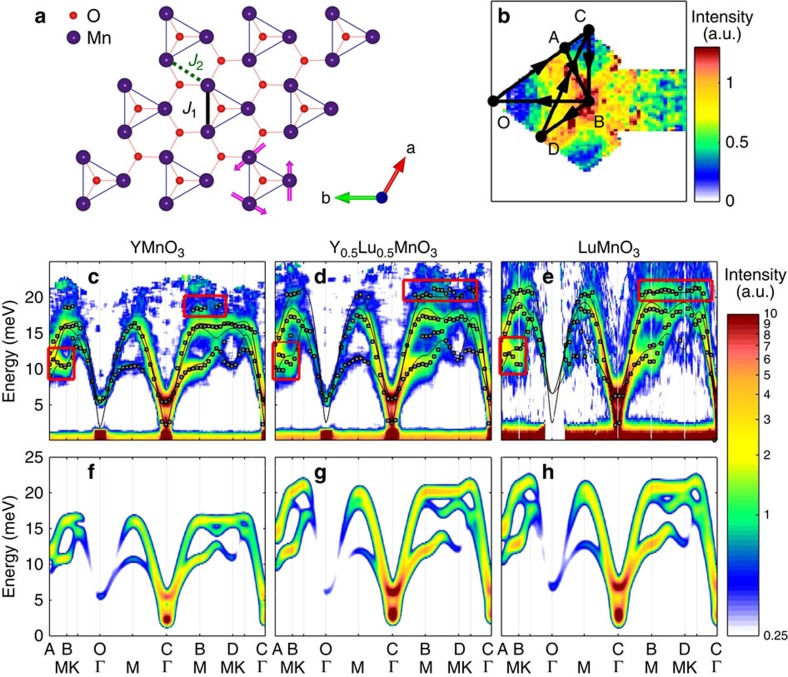
Magnetic excitation spectra in (Y,Lu)MnO_3_. (**a**) A Mn-O layer in RMnO_3_ forming a distorted 2D triangular antiferromagnet. (**b**) Inelastic neutron scattering data on LuMnO_3_ summed over an energy window of [19.5, 20.5] meV. The arrows in **b** indicate the reciprocal points where the data shown in **c**–**e** are cut. (**c**–**e**) The inelastic neutron-scattering data along the high symmetric directions (red circle and contour map) and fitted dispersion (black solid curve) for (**c**) YMnO_3_, (**d**) Y_0.5_Lu_0.5_MnO_3_ and (**e**) LuMnO_3_ calculated by linear spin wave theory. (**f**–**h**) Calculated dynamical spin structure factors using the minimal spin Hamiltonian, equation (4) in the Supplementary Note 2 for (**f**) YMnO_3_, (**g**) Y_0.5_Lu_0.5_MnO_3_ and (**h**) LuMnO_3_. For our simulations, we used the momentum and energy resolution of the instrument at the elastic line.

Hexagonal rare-earth manganite RMnO_3_ represents a good model system for geometrical frustration on a 2D triangular lattice: the nearest-neighbour antiferromagnetic interaction between *S*=2 (Mn^3+^) spins dominate, whereas the interlayer interaction is relatively weak^13^,^14^. We should note that it also exhibits a very large spin–lattice coupling when it becomes magnetically ordered^5^. In a more recent work, we reported experimental evidence for a spontaneous magnon decay and a remarkably large spatial anisotropy for Mn^3+^ ions in the exchange interactions for LuMnO_3_. This was attributed to a structural distortion in which groups of three Mn atoms become more closely bound^9^, such that the intratrimer *J*_1_ exchange constants may differ from the intertrimer *J*_2_ (see Fig. 1). Similar interpretations were shared by other groups too^15^,^16^. However, we should note that the large *J*_1_/*J*_2_ ratio of 6.4 obtained from fitting the data is inconsistent with the value of 1.15, obtained from first-principles calculations^17^ that used the experimental atomic positions as reported by neutron diffraction^5^. This realization prompted us to handle both magnon and phonon, as well as their cross-coupling on an equal footing and to go beyond the linear spin wave analysis.

Figure 1 shows the spin waves measured at the MAPS beamline of the ISIS facility together with the theoretical dispersion relation calculated from the spin Hamiltonian by a linear spin wave theory using the following parameters (see Supplementary Note 2): for YMnO_3_: *J*_1_=4, *J*_2_=1.8, *D*_1_=0.28, *D*_2_=−0.02 meV; for Y_0.5_Lu_0.5_MnO_3_: *J*_1_=12.5, *J*_2_=0.97, *D*_1_=0.18, *D*_2_=−0.018 meV; for LuMnO_3_: *J*_1_=9, *J*_2_=1.4, *D*_1_=0.28, *D*_2_=−0.02 meV. Despite the apparent success of the linear spin wave calculations, there lies a critical failure: first, the downward curvature along the AB direction and, second, the additional peaks at ∼19 meV indicated by a red box in Fig. 1c–e. However, most importantly, here we have to use an unphysically large *J*_1_/*J*_2_ ratio, to explain the additional high-energy peaks. Apart from the large *J*_1_/*J*_2_ ratio, this analysis of the linear spin waves has another drawback: which is that the calculated dynamical structure factor using the linear spin wave theory as shown in Fig. 1f–h always produces stronger intensity at the top mode of the spin waves than at the middle one, in marked contrast with the experimental data.

This discrepancy requires us to adopt a radically different approach to explain the measured full spin waves and go beyond the standard linear spin wave theory. One clue for how to address this problem can be taken from the physical properties: for example, our earlier neutron diffraction data revealed a giant spin–lattice coupling for (Y,Lu)MnO_3_ (ref. 5). This observation was subsequently corroborated by independent measurements on rare-earth hexagonal RMnO_3_ (refs 18, 19, 20, 21, 22, 23, 24, 25). More importantly, ultrasound measurements on YMnO_3_ found marked softening in C_11_ and C_66_, supporting our view that there is a strong in-plane deformation below *T*_N_^26^. This observation naturally indicates the importance of magnon and in-plane phonon coupling. This conclusion is also backed up by theoretical calculations^17^,^27^.

### Calculation of magneto-elastic excitations

Following this idea of a large spin–lattice coupling in RMnO_3_, we took a first-principles approach to the magnon–phonon coupling. First, we construct the following full model Hamiltonian, which couples the in-plane manganese vibrations directly to the spin system:


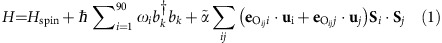


where 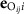 denotes the unit vector connecting the *i*-th manganese atom and the neighbouring oxygen atoms between the *i*-th and *j*-th manganese atoms as shown in Supplementary Fig. 2, 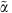 is the exchange striction, 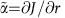, which is naturally made into a dimensionless exchange–striction constant 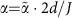
28, and *d* is the Mn–O bond length at the equilibrium. Therefore, our Hamiltonian takes into account the modulations of the Mn–O bond length as a function of Mn displacements.

Before going into detailed discussion, we would like to make a general remark on the related issue. In cases when the spin rotational symmetry is broken completely in the ground state, that is, when the spin structure is non-collinear, the Heisenberg term of the Hamiltonian provides a coupling of the transverse and longitudinal modes on neighbouring sites. That is, in terms of the local site-dependent preferred spin direction of the ordered state, the coupling terms take the form of the type 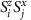 and so on. In the magnon language, they are quantized into the ‘odd' terms, producing linear 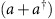 and cubic (*a*^†^*aa* and so on) contributions. In equilibrium, the linear magnon term must vanish, leaving the anharmonic cubic magnon coupling to be the sole outcome, which is important for magnon decays. However, in the presence of coupling to phonons, the linear 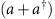 terms is ‘activated', as the local atomic displacements (*u*_*i*_) violate the equilibrium conditions locally, hence the ‘direct' coupling of magnons and phonons.

**Figure 2:**
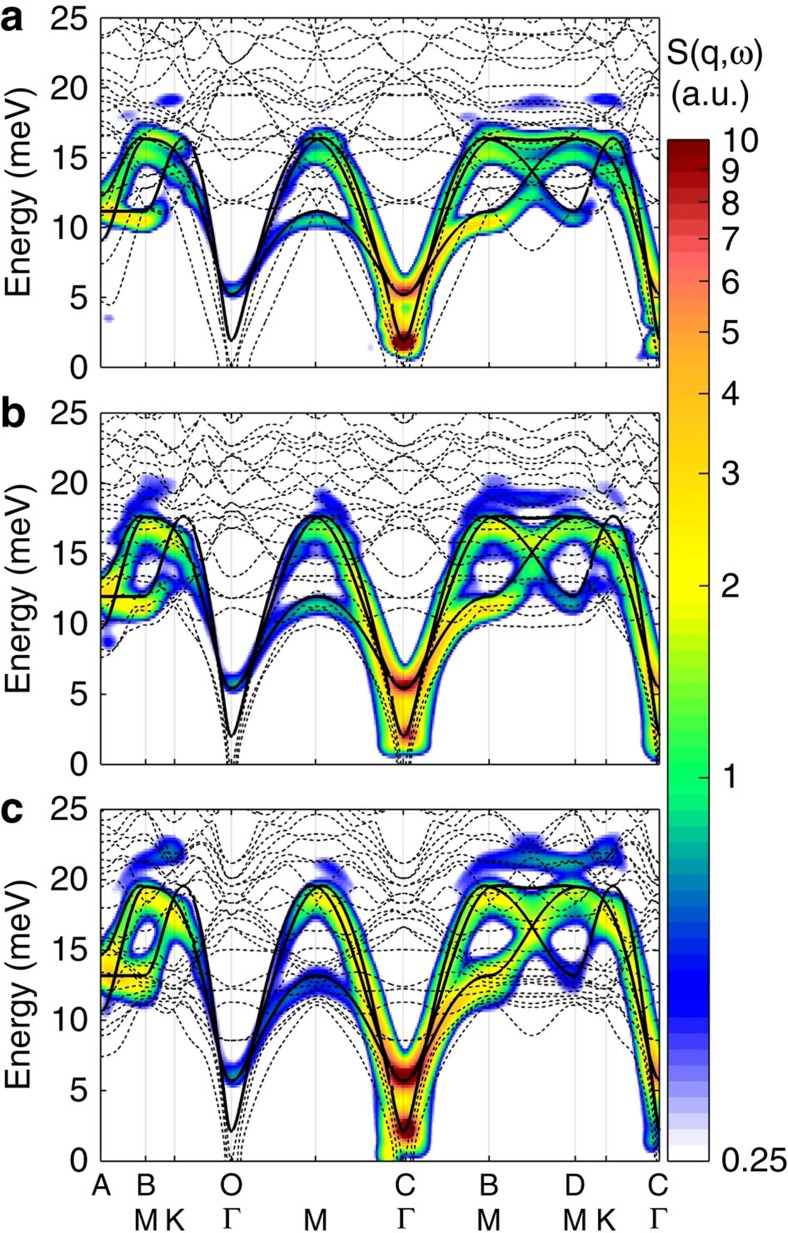
Calculated dynamical structure factor of magneto-elastic excitation. The dynamical spin structure factor calculated from the full Hamiltonian, equation (1) (contour map) by taking into account the magnon–phonon coupling: the phonon dispersion curves (dashed lines) and the magnon dispersion without the coupling (solid lines) for (**a**) YMnO_3_, (**b**) Y_0.5_Lu_0.5_MnO_3_ and (**c**) LuMnO_3_.

To calculate the full dispersions of all 90 phonon modes for the unit cell with six formula units, we used a first-principles density functional theory (DFT). We show the full phonon dispersion curves for the three compounds as dashed lines in Fig. 2. We note that the calculated phonon density of states (DOS) is in good agreement with the phonon spectra we measured using powder YMnO_3_ and LuMnO_3_ at the AMATERAS beamline of J-PARC (see Supplementary Fig. 1). We then calculated the dynamical spin structure factor within the linear approximation by using the full Hamiltonian above with the explicit magnon–phonon coupling: we used the dimensionless exchange–striction coefficients of *α*∼16–20.

The exchange–striction constant can also be estimated by using the pressure dependence of the crystal structure and the antiferromagnetic transition reported for YMnO_3_ (refs 29, 30). Using the experimental data reported in refs 9, 10, we came to an estimate of the dimensionless exchange striction *α* of 14 with the following formula: 
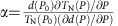
. Here, *d* is average Mn–O bond length, which is approximately one-third of lattice constant *a*. The experimental parameters used in our estimate are 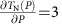 K GPa^−1^ (ref. 29) and 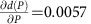 Å GPa^−1^ (ref. 30). Below we point out that our own data for the magnon excitation spectrum imply the value of the magneto-striction of the same order. This should be contrasted with the cuprates family, where estimates for an equivalent quantity are substantially smaller, *α*∼2–7(refs 31, 32).

By comparing with the experimental data, we obtained the best fitting results with the following sets of the parameters: *J*_1_=*J*_2_=2.5 meV, *D*_1_=0.28 meV, *D*_2_=−0.02 meV and *α*=16 for YMnO_3_; *J*_1_=*J*_2_=2.7 meV, *D*_1_=0.28 meV, *D*_2_=−0.02 meV and *α*=20 for Y_0.5_Lu_0.5_MnO_3_; and *J*_1_=*J*_2_=3 meV, *D*_1_=0.28 meV, *D*_2_=−0.02 meV and *α*=16 for LuMnO_3_ (see Supplementary Table 1). We note that according to the DFT calculations^17^ the relative difference between *J*_1_ and *J*_2_ is theoretically about 10–20% at maximum. Therefore, we judge that this choice of *J*_1_=*J*_2_ in our analysis is good enough to capture the essential underlying physics of (Y,Lu)MnO_3_, which is the magnon–phonon coupling.

The results shown as colour contour plot in Fig. 2 reproduce the overall features of the observed spectra of the experimental data in Fig. 1. It clearly shows that the high-energy signals located at 18∼20 meV come from a direct coupling between the magnon and the optical phonons, that is, a magneto-elastic mode. The intensity of this high energy magneto-phonon modes becomes stronger for Lu-enriched compounds due to the larger Mn phonon DOS at this energy for the Lu-enriched compounds, consistent with the experimental results shown in Fig. 1. This conclusion on the relevance of the magnon–phonon coupling for RMnO_3_ is also supported by the fact that our calculated spin waves successfully explains the downward curvature of the bottom magnon branch along the AB direction. In fact, an estimate of the exchange striction from the splitting of the high-energy hybrid modes in Fig. 2 yields the values in the same range, *α*∼10–20 (see Supplementary Note 3). We also note that our polarized neutron-scattering data are in good agreement with our calculations (see Supplementary Fig. 4 and Supplementary Note 4).

### Spontaneous decay of the hybrid mode

**Figure 3:**
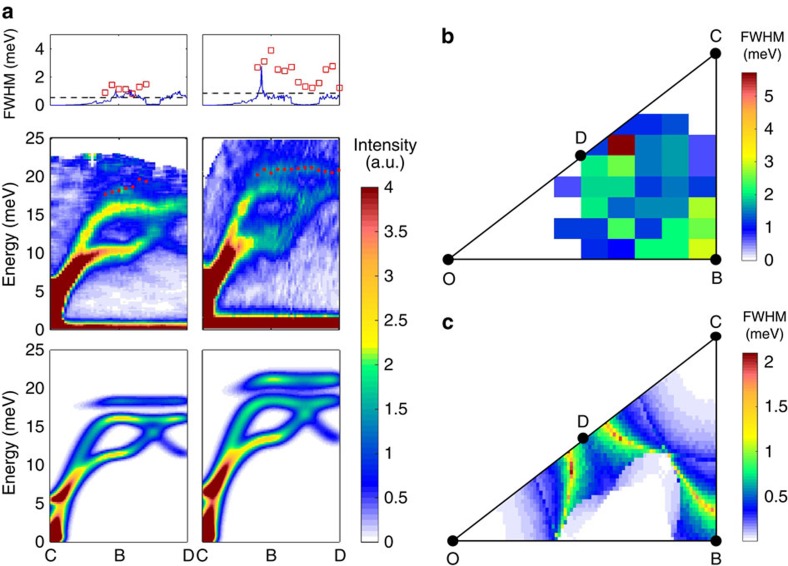
Linewidth broadening of magneto-elastic excitation. (**a**) The neutron-scattering data along the *CBD* direction (middle) and the calculated dynamical structure factor within the linear spin wave theory (bottom). Observed linewidth broadening of the top mode (square) together with the calculated result from the 1/*S* approximation (line) and the experimental resolution (dashed line) (top) for YMnO_3_ (left) and LuMnO_3_ (right). (**b**) Observed linewidth of the top mode for LuMnO_3_ (contour map) and (**c**) calculated intrinsic broadening of the top mode using the model Hamiltonian, equation (2). The experimental linewidth of the top mode was estimated by using multi-Gaussian functions and the typical results are shown in Supplementary Fig. 3.

In addition to the magnon–phonon hybridization, non-collinear spin structures allow three magnon interactions as discussed above, which can lead to spontaneous magnon decay into two magnon states when the kinematic conditions are satisfied. The magneto-elastic excitations have, by definition, both magnon and phonon characters. Therefore, the above mechanism can also lead to the decay of magneto-elastic excitations inside the two quasi-particles continuum of magnon. Indeed, we observe significant broadening of the top mode in LuMnO_3_ near the *B* and *D* points as shown in Fig. 3 and Supplementary Fig. 3: less strong broadening has been seen for other two compounds.

To calculate the decay rate directly and compare it with the experimental data, we simplify the problem by assuming a dispersionless optical phonon mode at ∼20 meV, where the strongest coupling has been observed in our data. Next, our model Hamiltonian reads as follows:


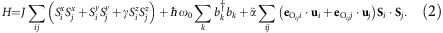


First, we calculate the dynamical spin structure factor by using a standard method. As shown in Fig. 3a, despite the simplification, the calculation results reproduce well the experimental intensity along the *C*–*B*–*D* direction. For the calculations, we used the following set of the parameters: for YMnO_3_: *J*=2.7 meV, *γ*=0.93, *ħω*_*0*_=17.5 meV, *α*=7.2; for LuMnO_3_: *J*=3.2 meV, *γ*=0.93, *ħω*_0_=19.5 meV, *α*=8. Next, the decay rate of the high-energy mode was calculated using an anharmonic spin wave theory within the 1/*S* approximation. The calculated results summarized in Fig. 3 also show the significant linewidth broadening for the top mode near the *B* and *D* points only for LuMnO_3_, consistent with the experimental results.

The reason for this is that in LuMnO_3_, the combination of the higher energies of the magnon and magnetoelastic modes means that more decay channels, including two quasiparticle emission, are kinematically allowed. The different decay channels have different boundaries in the reciprocal space, which also corresponds to logarithmic singularities in the decay rate^33^, and the largest broadening is observed at momentum transfers where the single-quasiparticle dispersion crosses these boundaries, such as at the *B* point. In the case of YMnO_3_, no such crossing occurs; thus, there are fewer decay channels available explaining why the observed linewidths remain narrow. Here we should stress that the single-magnon branches do not cross the line of singularities, whereas the magnetoelastic mode does; thus, a pure magnon decay is forbidden in this case. Similarly, the intrinsic decay rate of phonons is usually small due to a weak cubic anharmonicity. Thus, the strong hybridization of magnons and phonons provides a new mechanism to enhancing the magnon decays.

## Discussion

Our studies using an exchange-striction model indicates that the deviations from the linear spin wave theory must be a common feature for other triangular antiferromagnets with a non-collinear magnetic order. Looking beyond the 2D triangular antiferromagnets, we believe that the idea of magnon–phonon coupling can also be important in the studies of a wide variety of the 3*d* transition-metal magnetic compounds. For example, similar analysis might shed a light on the investigation of spin phonon coupling mechanism and anharmonic effects in many other important non-collinear magnets that exhibit a rather large spin–lattice coupling, such as spinel^34^ and invar materials^35^, which should be dominated by the exchange–striction as discussed in ref. 36. This is in contrast to the previous studies on magnon–phonon coupling focusing on materials with strong spin–orbit coupling such as rare-earth elements^37^.

To summarize, we mapped out the spin waves and phonon excitations of (Y,Lu)MnO_3_ over the Brillouin zone. By carrying out the spin wave calculations using the full Hamiltonian with both magnons and phonons on an equal footing and their coupling, we have not only demonstrated in our inelastic neutron-scattering data a clear sign of magnon–phonon coupling, but also have quantified the coupling strength directly. Our work provides the rare experimental test and quantification of magnon–phonon coupling in real materials and opens a new window of opportunities in other materials such as 2D triangular lattice and other frustrated systems, where such couplings, hitherto hidden, have been long suspected.

## Methods

### Sample preparation

We synthesized powder samples using a solid-state reaction method following the recipe as described in the literature^38^. We then grew single crystals of Y_1−*x*_Lu_*x*_MnO_3_ (with typical size of 5 × 5 × 40 mm^3^) by using a commercial optical floating zone furnace (Crystal Systems, Japan). Our subsequent powder and single crystal XRD confirmed that all our samples are prepared in high quality. We also measured the bulk properties (susceptibility and heat capacity) of all the samples to further confirm the quality by using a commercial set-up (MPMS5XL and PPMS9, Quantum Design USA).

### Inelastic neutron scattering

Inelastic neutron-scattering experiments have been performed on single crystal samples using the MAPS time-of-flight spectrometer at ISIS, UK, and a triple axes spectrometer with polarization analysis at Chalk River, Canada. In the time-of-flight experiments, incident energies were chosen at 40 meV for LuMnO_3_, 35 meV for Y_0.5_Lu_0.5_MnO_3_ and 30 meV for YMnO_3_ to adjust to the slight variations in the energy scales for each samples. The chopper frequency has been set to 250 Hz, which gave us a full width at half maximum energy resolution of 0.43∼1.36 meV depending on the energy transfer. The measurements have been performed at 4 K for YMnO_3_ and Y_0.5_Lu_0.5_MnO_3_, and 13 K for LuMnO_3_. We used the Horace programme for our data analysis^39^. In the triple axes spectrometer experiments, spin-polarized neutrons have been produced by using vertical focusing Heusler monochromator and analyser with the final energy fixed at 13.7 meV. To measure phonon DOS, inelastic neutron-scattering experiments have also been performed on the powder samples with the incident energy of 42 meV using the AMATERAS beamline at J-PARC, Japan.

### Theoretical calculations

We carried out first-principles calculations of phonon using a DFT+U method with *U*=4 eV. We used the PHONOPY code based on the force constant method^40^. In addition, the force constants were constructed by means of a supercell approach based on the density functional perturbation theory^41^, implemented in the VASP code^42^. Detailed discussion is given in the Supplementary Note 1.

For the spin waves calculations, we used a rotating framework with the direction of easy axis anisotropy being rotated from parallel to perpendicular to the crystallographic axes. To make our calculations simpler and transparent, we ignored the interlayer exchange coupling as it is known to be more than 100 times smaller than the in-plane coupling^9^,^14^. Using this approximation, we can have the following minimal Hamiltonian:


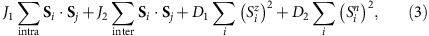


where *J*_1_ and *J*_2_ represent intra- and inter-trimer exchange constants and *D*_1_ and *D*_2_ are two magnetic anisotropies. We then calculated the spin wave dispersion using the standard linear spin wave theory^43^. We give detailed description of our spin wave calculations for the full Hamiltonian with the magnon and phonon coupling and the magnon–magnon nonlinear interaction in the Supplementary Notes 2 and 3.

### Data availability

All relevant data that support the findings of this study are available from the corresponding author on request.

## Additional information

**How to cite this article:** Oh, J. *et al*. Spontaneous decays of magneto-elastic excitations in non-collinear antiferromagnet (Y,Lu)MnO_3_. *Nat. Commun.* **7,** 13146 doi: 10.1038/ncomms13146 (2016).

## Supplementary Material

Supplementary Information Supplementary Figures 1-4, Supplementary Table 1, Supplementary Notes 1-4 and Supplementary References
