# Supplementary material for: Spontaneous decays of magneto-elastic excitations in non-collinear antiferromagnet (Y,Lu)MnO3
Source: Nat Commun. 2016 Oct 19;7:13146. doi: 10.1038/ncomms13146 (PMC5075801; doi:10.1038/ncomms13146)
Supplement: Supplementary Information — Supplementary Figures 1-4, Supplementary Table 1, Supplementary Notes 1-4 and Supplementary References [file ncomms13146-s1.pdf]

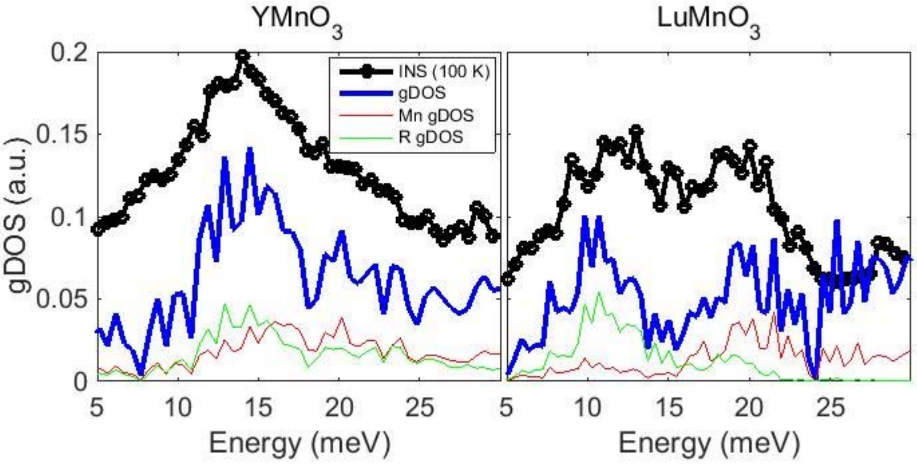

Supplementary Figure 1 **Phonon density of states (DOS)**. It compares the measured experimental phonon DOS with the theoretical results: the inelastic neutron scattering data taken at 100 K (black), the calculated gDOS (blue), manganese gDOS (red) and rare-earth elements gDOS (green).

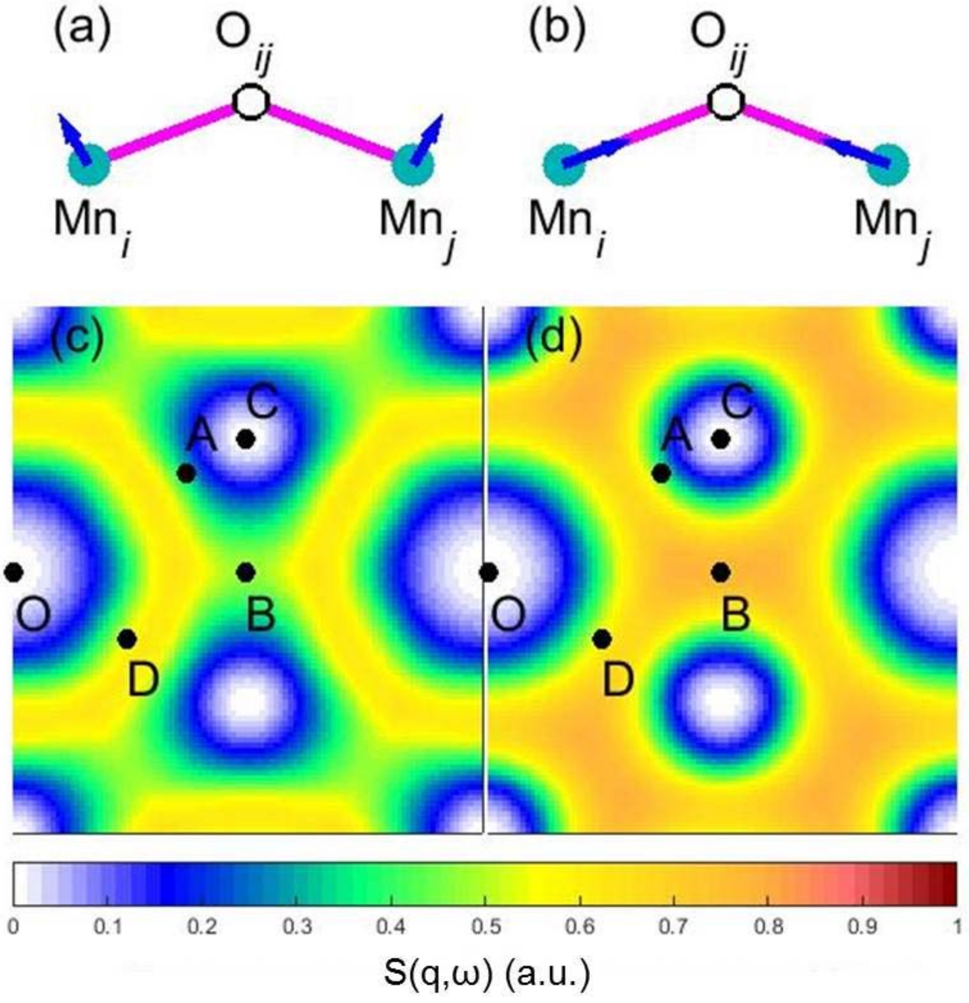

Supplementary Figure 2 **Q-dependent dynamical structure factor of the magneto-elastic mode.** It depicts the manganese displacement for the two cases, where there are variations in (a) a bond angle and (b) a bond length, respectively. The q-dependent dynamical structure factor of the high energy magnetoelastic mode calculated using Eq. (14) is given for the case of the structural modulation in (c) a bond angle change and (d) a bond length change. We note that our calculated dynamical structure factor for a scenario of bond-angle modulation (a & c) fails to reproduce the experimental momentum dependence shown in Fig. 1(b) as compared with those for a scenario of bond-length modulation (b & d).

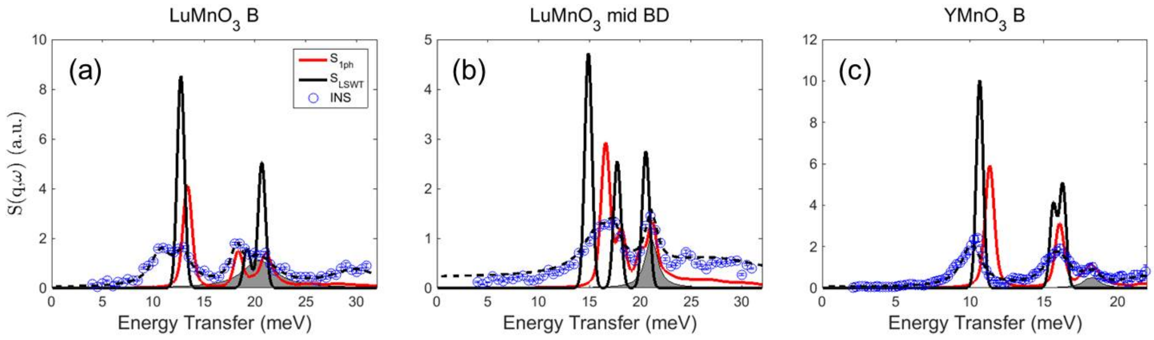

Supplementary Figure 3 **Comparison of experimental and theoretical results.** Constant q-cuts (circle) along the BD direction compared to the two theoretical cases: one is the 1/S expansion results for one phonon model using Eq. (14) (red line) and the other is LSWT without magnon phonon coupling calculated using Eq. (4) (black line) for (a,b) $LuMnO_3$ and (c) $YMnO_3$. The dashed line shows the fitting results using multi Gaussian functions, and the shaded area indicates the linewidth broadening of the magneto-elastic mode. Broad signals at high energy are attributed to two magnon continuum.

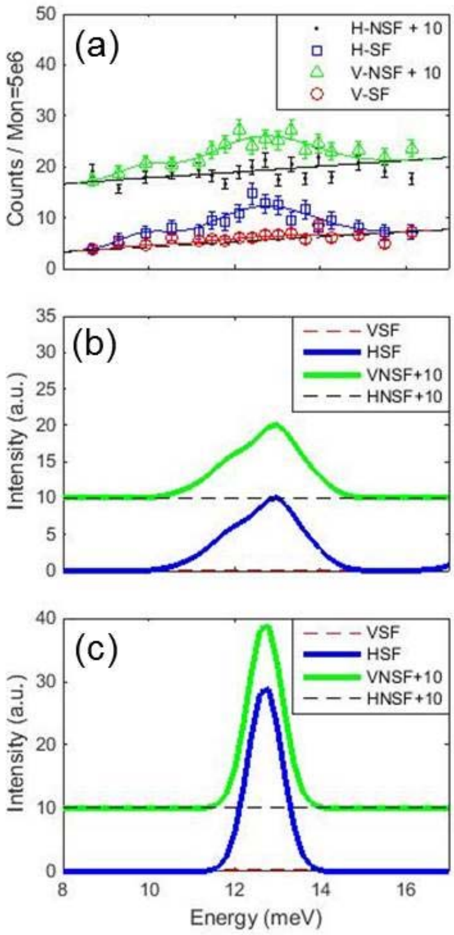

Supplementary Figure 4 **Polarized neutron spectra at the B point in $LuMnO_3$.** (a) The measured spectra are shown for the following four cases: for the vertical field non-spin flip channel (green), the horizontal field non-spin flip channel (black), the horizontal field spin flip channel (blue) and the vertical field spin flip channel (red). The error bars are the standard deviation of each data points experimentally measured. (b) The calculated spectra with the magnon phonon coupling using the first-principle phonon result. (c) The calculated spectra without the magnon phonon coupling.

	J_1 (meV)	J_2 (meV)	D_1 (meV)	D_2 (meV)	α
$YMnO_3$	4	1.8	0.28	-0.02	0
	2.5	2.5	0.28	-0.02	16
$Y_{0.5}Lu_{0.5}MnO_3$	12.5	0.97	0.18	-0.018	0
	2.7	2.7	0.28	-0.02	20
$LuMnO_3$	9	1.4	0.28	-0.02	0
	3	3	0.28	-0.02	16

Supplementary Table 1 **Summary of parameters for models with ($\alpha \neq 0$) and without ($\alpha = 0$) magnon phonon coupling**

Supplementary Note 1. Phonon calculation

We calculated the full phonon dispersion curves for $Y_{1-x}Lu_xMnO_3$ using the PHONOPY code [1] based on the force constant method [2]. The force constants were constructed by means of a supercell approach based on the density functional perturbation theory (DFPT) [3], implemented in the VASP code. It is well-known that the highly-localized levels such as Mn-3d orbital are not well described by normal DFT calculations, and thus we applied the Perdew Burke-Ernzerhof (PBE) method [4] including U in order to restore the strong electron-electron exchange-correlation effect [5]. This method was employed for the better description of the Mn-3d orbital in $RMnO_3$. Specifically, we used the value of U for the 3d orbital of Mn as 4 eV, which is slightly larger than 2.6 eV estimated from previous calculations [6]. Then, we constructed a rhombohedral-shape supercell, corresponding to 3 unit cells containing 90 atoms. Although manganese spins couple antiferromagnetically, we used a ferromagnetic spin structure to preserve the crystal symmetry. Another way to preserve the crystal symmetry is neglecting the spin character of Mn^{3+} spin, but the associated energy error is much larger for the latter [7]. All the atomic positions were fully relaxed until the Hellmann-Feynman force on each atom was reduced to 0.01 eV/Å. The calculated phonon DOS reproduces the peak structures well in the measured powder phonon spectra of $YMnO_3$ and $LuMnO_3$. We also checked that our calculated phonon dispersion along the [H 0 12] direction for $YMnO_3$ is in a good agreement with the result reported in Ref. [8].

Supplementary Note 2. Calculation of spin waves with a magnon phonon couplingPhonon

The phonon Hamiltonian and the displacement vector of the j-th atom in the l-th unit cell are given by:

$$H_{\mathrm{ph}} = \sum_k X_{\mathrm{ph}}^{\dagger}(k) \begin{pmatrix} \Omega & 0 \\ 0 & \Omega \end{pmatrix} X_{\mathrm{ph}}(k) \qquad (1)$$

$$X_{\mathrm{ph}}(k) = \left(b_{k1} \cdots b_{k\lambda} \cdots b_{-k1}^{\dagger} \cdots b_{-k\lambda}^{\dagger} \cdots\right)^{\mathrm{T}}, \Omega = \begin{pmatrix} \frac{\omega_{k1}}{2} & & 0 & \\ & \ddots & & \\ 0 & & \frac{\omega_{k1}}{2} & \\ & & & \ddots \end{pmatrix} \qquad (2)$$

$$\mathbf{U}_{j,l} = \sqrt{\frac{\hbar}{2Nm_j\omega_{k\lambda}}} \sum_{k,\lambda} \mathbf{V}_{j,k\lambda} e^{ik\cdot(R_l+r_j)}\left(b_{k\lambda} + b_{-k\lambda}^{\dagger}\right), \qquad (3)$$

where R and r indicate the unit cell and atom positions, while λ is a band index. The phonon frequencies $\omega_{k\lambda}$ and eigenvectors $\mathbf{V}_{j,k\lambda}$ have been calculated as described in the section 1 for the phonon calculations.

Magnon

For our spin waves calculations, we used the following ground states from Ref. [9]: Γ_3 for $YMnO_3$, Γ_4 for $LuMnO_3$, and Γ_3+Γ_4 for $(Y_{0.5}Lu_{0.5})MnO_3$. The spin Hamiltonian is given by:

$$H_\mathrm{m} = J_1 \sum_\mathrm{intra} \mathbf{S}_i \cdot \mathbf{S}_j + J_2 \sum_\mathrm{inter} \mathbf{S}_i \cdot \mathbf{S}_j + D_1 \sum_i (S_i^z)^2 + D_2 \sum_i (S_i^n)^2 \qquad (4)$$

Here, n denotes the direction of easy axis anisotropies, which is different for each of the three compounds. For the 120° magnetic structure, the spin Hamiltonian can be written in the following form using Holstein Primakoff operators:

$$H_\mathrm{m} = S \sum_k X_\mathrm{m}^\dagger (k) \begin{pmatrix} L & M \\ M & L \end{pmatrix} X_\mathrm{m}(k) \qquad (5)$$

$$\mathrm{L} = \begin{pmatrix} P_1 + \Delta I_3 & 0 \\ 0 & P_2^* + \Delta I_3 \end{pmatrix},\ M = \begin{pmatrix} 3P_1 + D_1 I_3 & 0 \\ 0 & 3P_2^* + D_1 I_3 \end{pmatrix}$$

$$P_1 = \begin{pmatrix} 0 & A_{12}^* & A_{31} \\ A_{12} & 0 & A_{23}^* \\ A_{31}^* & A_{23} & 0 \end{pmatrix},\ P_2 = \begin{pmatrix} 0 & A_{45}^* & A_{64} \\ A_{45} & 0 & A_{56}^* \\ A_{64}^* & A_{56} & 0 \end{pmatrix},\ I_3 = \begin{pmatrix} 1 & 0 & 0 \\ 0 & 1 & 0 \\ 0 & 0 & 1 \end{pmatrix} \qquad (6)$$

$$X_\mathrm{m}(k) = \left(a_{k1} \cdots a_{k6}\ a_{-k1}^\dagger \cdots a_{-k6}^\dagger\right)^\mathrm{T}$$

$$A_{lm} = \frac{1}{8} \sum_{i(l),j(m)} J_{ij} e^{ik\cdot(R(j)-R(i))},\ \Delta = \frac{1}{2}(J_1 + 2J_2 + D_1 - 2D_2) \qquad (7)$$

Here, $i(l)$ is the index of atoms belonging to the sublattice l and $R(i)$ is the unit cell position of such atoms.

Magnon-phonon coupling

The coupling term for an exchange-striction model is given as follows:

$$H_\mathrm{mp} = \tilde{\alpha} \sum_{ij} \left(\mathbf{e}_{\mathrm{O}_{ij}i} \cdot \mathbf{U}_i + \mathbf{e}_{\mathrm{O}_{ij}j} \cdot \mathbf{U}_j\right) \mathbf{S}_i \cdot \mathbf{S}_j \qquad (8)$$

Here, $\mathbf{U}_i$ is the displacement vector of i-th manganese atom, $\mathbf{e}_{\mathrm{O}_{ij}i}$ denotes the unit vector connecting the i-th manganese atom and the neighbouring oxygen atoms between the i-th and j-th manganese atoms as shown in Fig. SI2, $\tilde{\alpha}$ is the exchange striction, $\tilde{\alpha} = \partial J/\partial r$, which is naturally made into a dimensionless exchange-striction constant $\alpha = \tilde{\alpha} \cdot 2d/J$, and d is Mn-O bond length at the equilibrium. We ignore the oxygen vibrations due to a small oxygen DOS below 20 meV, where the magneto-elastic excitations is present. After substituting the displacement vectors and spin operators into phonon and Holstein Primakoff operators as described above, the total Hamiltonian becomes

$$H_{\mathrm{tot}} = S\sum_k X_{\mathrm{tot}}^{\dagger}\begin{pmatrix} L & -iN^{\dagger} & M & -iN^{\dagger} \\ iN & \Omega & -iN & 0 \\ M & iN^{\dagger} & L & -iN^{\dagger} \\ iN & 0 & iN & \Omega \end{pmatrix} X_{\mathrm{tot}} \qquad (9)$$

$$\mathrm{X}_{\mathrm{tot}} = \left(a_{k1} \cdots a_{k6}\; b_{k1} \cdots b_{k\lambda}\; \cdots\; a_{-k1}^{\dagger} \cdots a_{-k6}^{\dagger}\; b_{-k1}^{\dagger} \cdots b_{-k\lambda}^{\dagger} \cdots\right)^{\mathrm{T}} \qquad (10)$$

$$\mathrm{N}(\lambda,1) = \frac{1}{8}\sqrt{\frac{3\hbar S}{m_{\mathrm{Mn}}\omega_{k\lambda}}}\left(\mathbf{B}_{31}\cdot\mathbf{W}_{3,k\lambda}^{*} - \mathbf{B}_{21}\cdot\mathbf{W}_{2,k\lambda}^{*}\right)$$

$$\mathrm{N}(\lambda,2) = \frac{1}{8}\sqrt{\frac{3\hbar S}{m_{\mathrm{Mn}}\omega_{k\lambda}}}\left(\mathbf{B}_{12}\cdot\mathbf{W}_{1,k\lambda}^{*} - \mathbf{B}_{32}\cdot\mathbf{W}_{3,k\lambda}^{*}\right)$$

$$\mathrm{N}(\lambda,3) = \frac{1}{8}\sqrt{\frac{3\hbar S}{m_{\mathrm{Mn}}\omega_{k\lambda}}}\left(\mathbf{B}_{23}\cdot\mathbf{W}_{2,k\lambda}^{*} - \mathbf{B}_{13}\cdot\mathbf{W}_{1,k\lambda}^{*}\right)$$

$$\mathrm{N}(\lambda,4) = \frac{1}{8}\sqrt{\frac{3\hbar S}{m_{\mathrm{Mn}}\omega_{k\lambda}}}\left(\mathbf{B}_{64}\cdot\mathbf{W}_{6,k\lambda}^{*} - \mathbf{B}_{54}\cdot\mathbf{W}_{4,k\lambda}^{*}\right) \qquad (11)$$

$$\mathrm{N}(\lambda,5) = \frac{1}{8}\sqrt{\frac{3\hbar S}{m_{\mathrm{Mn}}\omega_{k\lambda}}}\left(\mathbf{B}_{45}\cdot\mathbf{W}_{4,k\lambda}^{*} - \mathbf{B}_{65}\cdot\mathbf{W}_{6,k\lambda}^{*}\right)$$

$$\mathrm{N}(\lambda,6) = \frac{1}{8}\sqrt{\frac{3\hbar S}{m_{\mathrm{Mn}}\omega_{k\lambda}}}\left(\mathbf{B}_{56}\cdot\mathbf{W}_{5,k\lambda}^{*} - \mathbf{B}_{46}\cdot\mathbf{W}_{4,k\lambda}^{*}\right)$$

and

$$\mathbf{B}_{lm} = \tilde{\alpha}\sum_{i(l),j(m)} \mathbf{e}_{\mathrm{O}_{ij}i}\, e^{ik\cdot(R(j)-R(i))} \qquad (12)$$

Here, $\mathbf{W}_{i,k\lambda} = \mathbf{V}_{i,k\lambda}e^{ik\cdot r_i}$. Then dynamical structure factors have been calculated following the standard method as discussed in Ref. [10]. By comparing with the experimental data, we obtained the best fitting results with the following sets of the parameters: J_1=J_2=2.5 meV, D_1=0.28 meV, D_2=-0.02 meV and α=16 for $YMnO_3$; J_l=J_2=2.7 meV, D_l=0.28 meV, D_2=-0.02 meV and α=20 for $Y_{0.5}Lu_{0.5}MnO_3$; J_l=J_2=3 meV, D_l=0.28 meV, D_2=-0.02 meV and α=16 for $LuMnO_3$. Note that the phonon energies of $Y_{0.5}Lu_{0.5}MnO_3$ have been increased by 15 % in order to reproduce the high energy additional peaks described in the main texts. It may be due to the error caused by the unrealistic Y and Lu positions of $Y_{0.5}Lu_{0.5}MnO_3$ we had to use in the first-principle phonon calculation to avoid the site dilution problem.

Supplemantary Note 3. Decay rate of magneto-elastic excitation

One phonon model

In the following Hamiltonian, we assumed one dispersionless optical phonon mode to form the magnon-phonon hybrid mode:

$$H = J\sum_{ij}\left(S_i^x S_j^x + S_i^y S_j^y + \tilde{\gamma} S_i^z S_j^z\right) + \hbar\omega_0 \sum_k b_{kx(y)}^\dagger b_{kx(y)} + \tilde{\alpha}\sum_{ij}\left(\mathbf{e}_{\mathrm{O}_{ij}i}\cdot\mathbf{U}_i + \mathbf{e}_{\mathrm{O}_{ij}j}\cdot\mathbf{U}_j\right)\mathbf{S}_i\cdot\mathbf{S}_j \quad (14)$$

Here, $\mathbf{e}_{\mathrm{O}_{ij}i}$ denotes a unit vector parallel or perpendicular to the line connecting i-th Mn atoms and O_{ij} depending on the spin-phonon coupling mechanism. After the successive application of Holstein Primakoff transformation and Bogoliubov transformation, the first term in the above equation can be summarized by the following equations:

$$J\sum_{ij}\left(S_i^x S_j^x + S_i^y S_j^y + \tilde{\gamma} S_i^z S_j^z\right) = S\sum_k A_k a_k^\dagger a_k - \frac{1}{2}B_k\left(a_k^\dagger a_{-k}^\dagger + a_{-k}a_k\right) = \sum_k \varepsilon_k \alpha_k^\dagger \alpha_k \quad (15)$$

$$\varepsilon_k = S\sqrt{A_k^2 - B_k^2},\ a_k = u_k\alpha_k + v_k\alpha_{-k}^\dagger,\ u_k^2 + v_k^2 = \frac{SA_k}{\varepsilon_k},\ 2u_k v_k = \frac{SB_k}{\varepsilon_k} \quad (16)$$

$$A_k = 3J\left(\left(\tilde{\gamma} - \frac{1}{2}\right)f_k + 1\right),\ B_k = \frac{3J}{2}(1 + 2\tilde{\gamma})f_k \quad (17)$$

$$f_k = \frac{1}{3}\sum_i \cos(\mathbf{k}\cdot\boldsymbol{\delta}_i) \quad (18)$$

$$\boldsymbol{\delta}_1 = (1,0),\ \boldsymbol{\delta}_2 = \left(-\frac{1}{2},\frac{\sqrt{3}}{2}\right),\ \boldsymbol{\delta}_3 = \left(-\frac{1}{2},-\frac{\sqrt{3}}{2}\right) \quad (19)$$

The quadratic part of the Hamiltonian can be written in the following matrix form:

$$H_2^k = Y^\dagger h Y \quad (20)$$

$$\mathrm{Y} = \left(\alpha_k\ \beta_k\ \alpha_{-k}^\dagger\ \beta_{-k}^\dagger\right)^{\mathrm{T}},\ h = \frac{1}{2}\begin{pmatrix} F & G \\ G & F \end{pmatrix} \quad (21)$$

$$\beta_k = (\chi_{kx}b_{kx} + \chi_{ky}b_{ky})/|\boldsymbol{\chi}_k| \quad (22)$$

$$F = \begin{pmatrix} \varepsilon_k & c_k \\ c_k & \hbar\omega_0 \end{pmatrix},\ G = \begin{pmatrix} 0 & c_k \\ c_k & 0 \end{pmatrix} \quad (23)$$

Here, the operator β_k represents a linear combination of the two degenerate phonon modes that is coupled to the magnon branch.

When the bond length change is important, the coupling factor $\boldsymbol{\chi}_k$ in (22) is given by

$$\boldsymbol{\chi}_k = \mathbf{e}_1\left(e^{i\mathbf{k}\cdot\boldsymbol{\delta}_2} - e^{-i\mathbf{k}\cdot\boldsymbol{\delta}_3}\right) + \mathbf{e}_2\left(e^{i\mathbf{k}\cdot\boldsymbol{\delta}_3} - e^{-i\mathbf{k}\cdot\boldsymbol{\delta}_1}\right) + \boldsymbol{e}_3\left(e^{i\mathbf{k}\cdot\boldsymbol{\delta}_1} - e^{-i\mathbf{k}\cdot\boldsymbol{\delta}_2}\right) \quad (24)$$

$$\mathbf{e}_1 = (0,1),\ \mathbf{e}_2 = \left(-\frac{\sqrt{3}}{2},-\frac{1}{2}\right),\ \mathbf{e}_3 = \left(\frac{\sqrt{3}}{2},-\frac{1}{2}\right) \quad (25)$$

Alternatively, when the bond angles are relevant, the coupling term is also given by eq. (20) with $\boldsymbol{\chi}_k = \boldsymbol{\delta}_2\left(e^{i\mathbf{k}\cdot\boldsymbol{\delta}_1} + e^{-i\mathbf{k}\cdot\boldsymbol{\delta}_3}\right) + \boldsymbol{\delta}_3\left(e^{i\mathbf{k}\cdot\boldsymbol{\delta}_2} + e^{-i\mathbf{k}\cdot\boldsymbol{\delta}_1}\right) + \boldsymbol{\delta}_1\left(e^{i\mathbf{k}\cdot\boldsymbol{\delta}_3} + e^{i\mathbf{k}\cdot\boldsymbol{\delta}_2}\right)$.

The magnon-phonon coupling parameter c_k in (23) can be written as

$$c_k = g \cdot JS\sqrt{\frac{3S}{8}}(u_k + v_k)|\boldsymbol{\chi}_k| \qquad (26)$$

where

$$g = \alpha \cdot \frac{1}{2d}\sqrt{\frac{\hbar}{2m_{\mathrm{Mn}}\omega_0}} \qquad (27)$$

Here, g is the product of the dimensionless exchange-striction strength α and the average zero-point atomic displacement in units of *2d* associated with the phonon mode ω_0: $\frac{1}{2d}\sqrt{\frac{\hbar}{2m_{\mathrm{Mn}}\omega_0}}$. Although the former constant is large, meaning that the dimensional exchange-striction $\tilde{\alpha} = \partial J/\partial r$ is several times larger than the exchange energy, the latter quantity is only about 0.015. Therefore, the overall magnon-phonon coupling strength still remains small compared to exchange energy. Using estimates for α above, altogether, $g{\sim}0.1 - 0.2$. This value of the magnon-phonon coupling is in agreement with the general discussion of it given in Refs. [11,12]. The smallness of the coupling also justifies neglecting the higher-order coupling terms as well as the more traditional two-magnon—one-phonon couplings as these lead to the effects of order g^2.

For the convenience of our calculations, we define the original magnon branches located below 20 meV as the "mode 0" and the top branches at about 20 meV as the "mode 1". Now, the Hamiltonian can be transformed to the diagonalized form [13] with the energies given by the following equations:

$$E_{0k} = \sqrt{\frac{\varepsilon_k^2+\hbar^2\omega_0^2-\sqrt{\left(\varepsilon_k^2+\hbar^2\omega_0^2\right)^2-4\left(\hbar^2\varepsilon_k^2\omega_0^2-4\hbar c_k{}^2\varepsilon_k\omega_0\right)}}{2}}$$

$$E_{1k} = \sqrt{\frac{\varepsilon_k^2+\hbar^2\omega_0^2+\sqrt{\left(\varepsilon_k^2+\hbar^2\omega_0^2\right)^2-4\left(\hbar^2\varepsilon_k^2\omega_0^2-4\hbar c_k{}^2\varepsilon_k\omega_0\right)}}{2}} \qquad (28)$$

We note that away from the level-crossing region, $\varepsilon_k \approx \hbar\omega_0$, Eq. (28) closely describes original magnon and phonon modes, but near that region they are strongly intermixed.

The additional Bogoliubov transformation that diagonalizes the Hamiltonian by mixing magnons and

phonons is

$$a_k = u_{0k}\gamma_{0,k} + u_{1k}\gamma_{1,k} + v_{0k}\gamma_{0,-k}^{\dagger} + v_{1k}\gamma_{1,-k}^{\dagger} \qquad (29)$$

where $\gamma_{0(1),k}$ are the operators of the new quasiparticles: magneto-phonon modes. Using the smallness of the magnon-phonon coupling parameter c_k in (26), associated with the smallness of the magnon-phonon coupling g, one can write the parameters of this transformation as:

$$u_{0k} \simeq u_k \Big/ \sqrt{1 + \left(\frac{c_k}{E_{0k} - \hbar\omega_0}\right)^2}$$

$$u_{1k} \simeq \left[\frac{c_k}{(E_{1k} - \varepsilon_k)} u_k - \frac{c_k}{(E_{1k} + \varepsilon_k)} v_k\right] \Big/ \sqrt{1 + \left(\frac{c_k}{E_{1k} - \varepsilon_k}\right)^2}$$

$$v_{0k} \simeq v_k \Big/ \sqrt{1 + \left(\frac{c_k}{E_{0k} - \hbar\omega_0}\right)^2}$$

$$v_{1k} \simeq \left[\frac{c_k}{(E_{1k} - \varepsilon_k)} v_k - \frac{c_k}{(E_{1k} + \varepsilon_k)} u_k\right] \Big/ \sqrt{1 + \left(\frac{c_k}{E_{1k} - \varepsilon_k}\right)^2} \qquad (30)$$

When the original, unperturbed magnon band is near the phonon branch, $\varepsilon_k \approx \hbar\omega_0$, the energies of the magneto-phonon modes can be written as

$$E_{0k} \simeq \varepsilon_k - \Delta_k$$

$$E_{1k} \simeq \hbar\omega_0 + \Delta_k$$

where the splitting

$$\Delta_k = c_k \qquad (31)$$

is *linear* in the magnon(spin)-phonon coupling. The physics here is that of the mode mixing and level repulsion. One can, therefore, be able to determine the dimensionless exchange-striction strength α directly from the value of the gap between the modes 0 and 1. Using the magnon bandwidth from Fig. SI3 of 20 meV, spin value S=2, and the splitting $2\Delta_k \approx 2$meV, an estimate from Eqs. (26,27) yields $\alpha \sim 10 - 20$, in agreement with the other approaches.

Cubic anharmonicity

The cubic term from the spin Hamiltonian is given by

$$H_3^0 \cong J\sqrt{\frac{S}{2}}\sum_{ij}\sin\theta_{ij}\left[a_i^\dagger a_i\left(a_j^\dagger + a_j\right) - a_j^\dagger a_j\left(a_i^\dagger + a_i\right)\right]$$

$$= iJ\sqrt{2S}\sum_{ij}\sum_{k_{1,2,3}}\sin\left(\mathbf{Q}\cdot\boldsymbol{\delta}_{ij}\right)\sin\left(\mathbf{k}_3\cdot\boldsymbol{\delta}_{ij}\right)\left(a_{-k_1}^\dagger a_{k_2} a_{k_3} + a_{-k_1}^\dagger a_{k_2} a_{-k_3}^\dagger\right) \qquad (32)$$

Here, $\mathbf{Q} = (4\pi/3a, 0)$ is the ordering vector associated with the 120° ordering structure.

Note that the cubic term from the spin phonon coupling is negligible due to small mean fluctuations of atoms as discussed in Section 2.5. Using Eq. (29), the symmetrized Hamiltonian becomes

$$H_3 = \sum_{kq,\mu\nu\eta}\left[\frac{1}{2!}\Gamma_1^{\mu\nu\eta}(q, k-q; k)\gamma_{\mu,q}^\dagger\gamma_{\nu,k-q}^\dagger\gamma_{\eta,k} + \frac{1}{3!}\Gamma_2^{\mu\nu\eta}(q, -k-q, k)\gamma_{\mu,q}^\dagger\gamma_{\nu,-k-q}^\dagger\gamma_{\eta,k}^\dagger\right] \quad (33)$$

$$\Gamma_{1,2}^{\mu\nu\eta} = 3iJ\sqrt{\frac{3S}{2}}\tilde{\Gamma}_{1,2}^{\mu\nu\eta} \qquad (34)$$

$$\tilde{\Gamma}_1^{\mu\nu\eta} = \bar{f}_1\left(u_{\mu1} + v_{\mu1}\right)\left(u_{\nu2}u_{\eta3} + v_{\nu2}v_{\eta3}\right) + \bar{f}_2\left(u_{\nu2} + v_{\nu2}\right)\left(u_{\mu1}u_{\eta3} + v_{\mu1}v_{\eta3}\right) - \bar{f}_3\left(u_{\eta3} + v_{\eta3}\right)\left(u_{\mu1}v_{\nu2} + v_{\mu1}u_{\nu2}\right)$$

$$\tilde{\Gamma}_2^{\mu\nu\eta} = \bar{f}_1\left(u_{\mu1} + v_{\mu1}\right)\left(u_{\nu2}v_{\eta3} + v_{\nu2}u_{\eta3}\right) + \bar{f}_2\left(u_{\nu2} + v_{\nu2}\right)\left(u_{\mu1}v_{\eta3} + v_{\mu1}u_{\eta3}\right) + \bar{f}_3\left(u_{\eta3} + v_{\eta3}\right)\left(u_{\mu1}v_{\nu2} + v_{\mu1}u_{\nu2}\right)$$

$$\bar{f}_i = \frac{1}{3}\sum_j\sin\left(\mathbf{k}_i\cdot\mathbf{d}_j\right) \qquad (35)$$

Here μ, ν, η are 0 or 1 indices of the magneto-phonon modes in Eq. (29).

The relevant decay channel for the quasiparticle with energy E_{1k} (magneto-phonon mode) should be

$$\Gamma_1^{001}(q, k-q; k)\gamma_{0,q}^\dagger\gamma_{0,k-q}^\dagger\gamma_{1,k} \qquad (36)$$

due to the kinematic condition. To investigate the decay rate of each mode, the decay channels should be specified. Following the same notation for the modes we used for Eq. (29), we can neglect the 0→{0,0} decay channel due to a large easy-plane anisotropy close to the critical value of 0.92, below which the decays are completely eliminated [15]. Similarly, 1→{1,1} and 1→{1,0} decays are also forbidden since two quasi-particles states of the decay products should lie at much higher energies. Therefore, 1→{0,0} decay is the only relevant channel and the calculated minimum of two quasi-particles continuum from mode 0 is clearly below mode 1 Then, the decay rate of mode 1 is given by the following equation.

$$\Gamma_{1,k} = \frac{1}{2}\mathrm{Im}\left(\sum_q \frac{\left|\Gamma_1^{001}(q,k-q;k)\right|^2}{E_{1,k}-E_{0,q}-E_{0,k-q}+i0}\right) \quad (37)$$

The calculated result shown in Fig. 3 in the main text shows clear signatures of singularity in the decay rate. Although the actual decay rate should be regularized by a proper self-consistent approach, the decay rate would remain significant [14,15].

Dynamical structure factor

The general spectral properties of branch μ can be obtained from the spectral function

$$A_\mu(q,\omega) = -\frac{1}{\pi}\mathrm{Im}G_\mu(q,\omega) \quad (38)$$

The Green function for branch μ is given by

$$G_\mu^{-1}(q,\omega) = \omega - E_{\mu q} - \Sigma_\mu(q,\omega) \quad (39)$$

Since we are interested in linewidth broadening due to decays, only the imaginary part of the decay rate is considered. Then, the on-shell self-energy is given by

$$\Sigma_\mu(q,\omega = E_{\eta,q}) = -i\sum_{p,\mu\nu}\frac{\pi}{2}\left|\Gamma_1^{\mu\nu\eta}(p,q-p;q)\right|^2\delta\left(E_{\eta,q}-E_{\mu,p}-E_{\nu,q-p}\right) \quad (40)$$

The dynamical structure factor can be calculated using the spectral function Eq. (38).

$$S(q,\omega) = \left(\delta_{\alpha\beta} - \frac{k_\alpha k_\beta}{k^2}\right)S_{\alpha\beta}(q,\omega) \quad (41)$$

Up to the $O(1/S)$ order, each component of dynamical structure factor is given by the following equations [16].

$$S_{x_0x_0}(q,\omega) = S_{z_0z_0}(q,\omega) = \frac{1}{4}\left(S_{xx}(q_-,\omega) + S_{xx}(q_+,\omega) + S_{zz}(q_+,\omega)\right)$$

$$S_{y_0y_0}(q,\omega) = S_{yy}(q,\omega) \quad (42)$$

Then, Eq. (42) can be rewritten by

$$S(q,\omega) = \left(1 - \frac{k_y^2}{k^2}\right)S_{y_0y_0}(q,\omega) + \left(2 - \frac{k_x^2+k_z^2}{k^2}\right)S_{x_0x_0}(q,\omega) \quad (43)$$

$$S_{xx}(q,\omega) \approx \sum_\mu \frac{S}{2}\left(u_{\mu,q} + v_{\nu,q}\right)^2 A_{11}^\mu(q,\omega)$$

$$S_{yy}(q,\omega) \approx \sum_\mu \frac{S}{2}\left(u_{\mu,q} - v_{\nu,q}\right)^2 A_{11}^\mu(q,\omega)$$

$$S_{zz}(q,\omega) = \frac{1}{2}\sum_{p,\mu\nu}\left(u_{\mu,p}v_{\nu,p-q} + v_{\mu,p}u_{\nu,p-q}\right)^2\delta\left(\omega - E_{\mu,p} - E_{\nu,p-q}\right) \quad (44)$$

The result summarized in Supplementary Figure 3 describes the high energy peak intensities as well as the linewidth broadening.

Supplementary Note 4. Polarized inelastic neutron scattering

Polarized inelastic neutron scattering experiment on two coaligned $LuMnO_3$ single crystals (size~5×5×15 mm^3 each) has been performed using the C5 triple axes spectrometer at Chalk River Laboratories, Canada. A vertically focusing Heusler and two pyrolytic graphite filters are used as an analyzer. A five coil assembly has been used to control the field direction, and the final energy has been fixed to 13.7 meV. We performed the polarization analysis of the bottom mode at the B point. The spin flip and nonspin flip channel for horizontal (**H**//**Q**) and vertical applied field (**H**//**c**) together with the calculation results are summarized in Fig. SI3. It shows that the spin phonon model explains the peak splitting of the bottom mode at B point as well as the polarization of the mode.

Supplementary References

1. Togo, A. & Tanaka, First principles phonon calculations in materials science. *Scr. Mater.* **108**, 1-5 (2015).
2. Parlinski, K., Li, Z. Q. & Kawazoe, Y. first-principles determination of the soft mode in cubic ZrO_2. *Phys. Rev. Lett.* **78**, 4063-4066 (1997).
3. Baroni, S., Gironcoli, S. de, Corso, A. Dal, & Giannozzi, P. Phonons and related crystal properties from density-functional perturbation theory. *Rev. Mod. Phys.* **73**, 515-562 (2001).
4. Perdew, J. P., Burke, K. & Ernzerhof, M. Generalized Gradient Approximation Made Simple. *Phys. Rev. Lett.* **77**, 3868 (1996).
5. Dudarev, S. L., Botton, G. A., Savrasov, S. Y., Humphreys, C. J. & Sutton, A. P. Electron-energy-loss spectra and the structural stability of nickel oxide: An LSDA+U study. *Phys. Rev. B* **57**, 1505-1509 (1998).
6. Solovyev, I. V., Valentyuk, M. V. & Mazurenko, V. V., Magnetic structure of hexagonal $YMnO_3$ and $LuMnO_3$ from a microscopic point of view. *Phys. Rev. B* **86**, 054407 (2012).
7. Varigon, J. *et al.* First principles calculation of the phonons modes in the hexagonal $YMnO_3$ ferroelectric and paraelectric phases. arXiv:1203.1752 (2012).
8. Petit, S., Moussa, F., Hennion, M., Pailhe`s, S., Pinsard-Gaudart, L., & Ivanov, A. Spin Phonon Coupling in Hexagonal Multiferroic $YMnO_3$. *Phys. Rev. Lett.* **99**, 266604 (2007).
9. Park, J., Lee, S., Kang, M., Jang, K-H., Lee, C., Streltsov, S. V., Mazurenko, V. V., Valentyuk, M. V., Medvedeva, J. E., Kamiyama, T., & Park, J.-G. Doping dependence of spin-lattice coupling and two-dimensional ordering in multiferroic hexagonal $Y_{1-x}Lu_xMnO_3$ ($0<x<1$). *Phys. Rev. B* **82**, 054428 (2010).
10. Petit, S. Numerical simulations and magnetism. *Ecoles Thématiques de la Société Française de la Neutronique* **12**, 105-121 (2011).
11. Chernyshev, A. L. & Brenig, W. Thermal conductivity in large-J two-dimensional anti ferromagnets: Role of phonon scattering. *Phys. Rev. B* **92,** 054409 (2015).
12. Bramwell, S. T. Temperature dependence of the isotropic exchange constant. *J. Phys. Condens. Matter* **2**, 7536 (1990).
13. White, R. M. *et al.*, Diagonalization of the Antiferromagnetic Magnon-Phonon interaction. *Phys. Rev.* **139**, A450-A454 (1965).
14. Chernyshev, A. L. & Zhitomirsky, M. E. Spin waves in a triangular lattice antiferromagnet: Decays, spectrum renormalization, and singularities. *Phys. Rev. B* **79**, 144416 (2009).
15. Zhitomirsky, M. E. & Chernyshev, A. L. Colloquium: Spontaneous magnon decays. *Rev. Mod.*

Phys. **85**, 219-242 (2013).

16. Mourigal, M., Fuhrman, W. T., Chernyshev, A. L. & Zhitomirsky, M. E. Dynamical structure factor of the triangular-lattice antiferromagnet. *Phys. Rev. B* **88**, 094407 (2013).
